# Forecasting mental states in schizophrenia using digital phenotyping data

**DOI:** 10.1371/journal.pdig.0000734

**Published:** 2025-02-07

**Authors:** Thierry Jean, Rose Guay Hottin, Pierre Orban

**Affiliations:** 1 Research Center of the Montreal Mental Health University Institute, Montreal, Canada; 2 Department of Psychiatry and Addictology, University of Montreal, Montreal, Canada; Liverpool John Moores University - City Campus: Liverpool John Moores University, UNITED KINGDOM OF GREAT BRITAIN AND NORTHERN IRELAND

## Abstract

The promise of machine learning successfully exploiting digital phenotyping data to forecast mental states in psychiatric populations could greatly improve clinical practice. Previous research focused on binary classification and continuous regression, disregarding the often ordinal nature of prediction targets derived from clinical rating scales. In addition, mental health ratings typically show important class imbalance or skewness that need to be accounted for when evaluating predictive performance. Besides it remains unclear which machine learning algorithm is best suited for forecast tasks, the eXtreme Gradient Boosting (XGBoost) and long short-term memory (LSTM) algorithms being 2  popular choices in digital phenotyping studies. The CrossCheck dataset includes 6,364 mental state surveys using 4-point ordinal rating scales and 23,551 days of smartphone sensor data contributed by patients with schizophrenia. We trained 120 machine learning models to forecast 10 mental states (e.g., Calm, Depressed, Seeing things) from passive sensor data on 2 predictive tasks (ordinal regression, binary classification) with 2 learning algorithms (XGBoost, LSTM) over 3 forecast horizons (same day, next day, next week). A majority of ordinal regression and binary classification models performed significantly above baseline, with macro-averaged mean absolute error values between 1.19 and 0.77, and balanced accuracy between 58% and 73%, which corresponds to similar levels of performance when these metrics are scaled. Results also showed that metrics that do not account for imbalance (mean absolute error, accuracy) systematically overestimated performance, XGBoost models performed on par with or better than LSTM models, and a significant yet very small decrease in performance was observed as the forecast horizon expanded. In conclusion, when using performance metrics that properly account for class imbalance, ordinal forecast models demonstrated comparable performance to the prevalent binary classification approach without losing valuable clinical information from self-reports, thus providing richer and easier to interpret predictions.

## Author summary

Symptoms associated with mental health disorders vary greatly over time. Periods of partial remission unfortunately alternate with relapses defined by a marked worsening of symptoms. Hence, assessing future risk and adopting preventive measures is a key challenge for clinical psychiatry. With their many sensors, smartphones can provide novel insights into human behavior outside the medical office. By using machine learning, a branch of artificial intelligence, it is possible to use such smartphone sensor data to predict future mental states and symptoms in psychiatric patients. The present work highlights the importance of predicting fine-grained levels of symptom severity, as commonly reported by patients using so-called ordinal rating scales. Such ordinal predictions were not less accurate than the simplified binary predictions (on/off, high/low) often reported in previous efforts. Besides, we underscore that severe mental states are rare compared to healthy ones, and that this imbalance brings methodological challenges that need to be taken into account to develop valid predictive models.

## Introduction

Although severe psychiatric disorders such as schizophrenia are often chronic, they are also notoriously temporally dynamic, with the severity of symptoms varying over time [1,2]. In a uniquely individual way, periods of partial remission alternate with recurrent relapses defined by a marked worsening of symptoms [3–5]. Monitoring a patient’s symptom trajectory and predicting future risks are therefore key clinical tasks in order to implement required preventive measures [6]. Unfortunately, routine medical appointments provide too few and distant observations to adequately monitor complex individual temporal dynamics [7,8]. Additionally, clinical information is primarily collected through interviews in the medical office, which has limited generalizability to the patient’s day-to-day life [9] and heavily depends on the partly flawed patient’s memory [10]. Digital phenotyping holds promise in this regard, as it allows continuously characterizing human behavior and mental health outside the medical environment using smartphones [11,12]. First, patients can use their device to periodically rate their symptoms on clinical scales as their daily life unfolds, the ability of remotely tracking symptoms over time leading to improved clinical outcomes [13]. Second, digital phenotyping leverages passive data from the device’s sensors (e.g., Wi-Fi, Bluetooth, GPS, accelerometer) to render rich facets of behavior. For instance, sedentariness may be associated with low GPS activity, and sleep disruption may be represented by nightly phone unlocks. Machine learning plays a key role in transforming this unprecedented volume and granularity of data into insights into mental health [14]. Critically, machine learning may be further exploited to develop models that accurately predict future fluctuating symptoms (e.g., frequency of hallucinations) and acute events (e.g., hospitalisations), which could improve clinical practice in the future [15–17].

Garcia-Ceja et al. [18] distinguish three types of studies that demonstrate the relevance of digital phenotyping to the characterization of mental illness: *association* studies merely explore the statistical relationships between inputs (e.g., sensor data) and a target (symptom level); *detection* studies use inputs to predict with machine learning the target at the current time, akin to diagnosis; and *forecasting* studies use inputs to predict with machine learning a target in the future, similar to prognosis. First, research validated the presence of statistical associations between passive sensing data and mental states, both in healthy [19] and clinical populations [20,21]. For instance, relevant digital markers can be established to differentiate healthy from clinical populations for accelerometry [22] and mobility [23,24] features, as highlighted in a review of 46 studies on this topic [25]. Second, several studies have demonstrated that mental health-related outcomes can be successfully predicted from smartphone sensor data using machine learning. In major depression, participants with an established diagnosis can be distinguished from non-depressed participants [26] and the absence or presence of specific symptoms can be predicted in this clinical population [27]. Similar works led to symptom-level detection in bipolar disorder [28] and schizophrenia samples [29]. The binary accuracy of such predictive models ranged from 65% to 98% across 40 studies [30]. The large majority of these studies used supervised machine learning, either classification or regression, with gradient-boosted decision trees, support vector machines, linear models, and neural networks being the most commonly used algorithms, in that order. Third, forecast studies providing predictions about future health outcomes have also been published, although they are scarcer than association and detection studies. The feasibility of predicting future mood and stress in a healthy population has been replicated a few times [31,32]. Similar approaches were successful in predicting clinical scale scores and specific psychiatric symptoms for depression [33], bipolar disorder [34], and anxiety [35]. The predictive task was either binary classification (i.e., low/high categories) or continuous regression (i.e., an outcome score), with the forecast horizon extending up to a week in the future. Most of the aforementioned forecast studies investigated the predictive performance of recurrent neural networks amongst other machine learning algorithms, in line with the idea that these types of algorithms are best suited for a forecasting task given their ability to model long-term dependencies and latent variables [36,37]. Despite their success in detection studies, gradient-boosted decision trees models were not thoroughly investigated for forecasting.

To date, detection and forecasting studies have focused on solving binary classification or continuous regression tasks even though the target of the prediction often comes from ordinal rating scales [18,25]. Consequently, the resulting binary or real-numbered predictions do not match the ordinal scale interpretation guidelines nor refer to well-defined constructs, leaving key clinical information behind. Previous work evaluated XGBoost models on the same dataset for the tasks of binary classification, continuous regression, and multiclass classification [38]. While multiclass classification preserves the original response items, it loses their ordering and faces the rank inconsistency problem [39]. Ordinal regression (or ordinal classification) models preserve classes and ordering resulting in rank-consistent discrete predictions easy to interpret with existing validated guidelines. Implicitly, binary classification and continuous regression are often used to mitigate the effect of the small number of examples per class (i.e., class imbalance) on performance. Alas, the data processing inequality from information theory states that variable transformations such as binarization cannot increase the variable’s information content [40,41]. Possible gains in predictive performance come at the cost of solving a problem that ignores nuances of the collected data. Still, transforming ordinal scale ratings into a binary target may be a well-motivated modelling decision if done based on a scale’s interpretation guidelines and not merely to simplify the predictive task or reduce class imbalance [26,42]. For all learning tasks, dedicated evaluation metrics are required to properly evaluate model performance when dealing with class imbalance [43].

Our first objective was to assess the potential performance cost of using ordinal regression compared to binary classification to forecast future mental states, using passive sensing data exclusively. We investigated the potential mediating effect of binarization on the relationship between class imbalance and performance. Our second objective was to provide a comprehensive benchmark of recurrent neural networks and gradient boosted decision trees models for digital phenotyping forecasting to question the implicitly assumed superiority of the former, while systematically exploring the effect of the forecast horizon on predictive performance. To this end, we used the publicly available digital phenotyping dataset CrossCheck [44,45]. Previous studies using these data have explored the relationship between passive sensing data and self-reported mental states [29,44–51], but none have specifically addressed the importance of preserving the ordinal nature of self-reports and the impact of class imbalance on performance.

## Methods

### Dataset

We obtained the publicly available de-identified data from the CrossCheck study released in 2020. This digital phenotyping dataset was collected as part of a randomized controlled trial (clinical trial registration: ClinicalTrials.gov, #NCT01952041) conducted at the Zucker Hillside Hospital in New York City, New York between 2015 and 2017. Ethics approval was obtained from the institutional review boards of Dartmouth College (#24356) and North Shore-Long Island Jewish Health System (#14-100B), and all psychiatric outpatients provided informed consent to participate. Inclusion criteria were a diagnosis of schizophrenia, schizoaffective disorder, or psychosis not otherwise specified; 18 years of age; a significant psychiatric event such as inpatient psychiatric hospitalization or psychiatric hospital emergency room visit within the last 12 months. Specific diagnoses were not included in the shared dataset. Data used in the present project comes from 62 patients assigned to the smartphone arm of the clinical trial. They were provided with a Samsung Galaxy S5 Android smartphone on which a mobile app continuously collected passively sensed data for up to one year.

A series of high-level passive sensing features were made available in the dataset (Table 1). Features were computed daily and separately for 6-hour periods: morning (6am–12pm), afternoon (12pm–6pm), evening (6pm–12pm) and night (12am–6am). In total, 23,551 days of passive sensing data, without any missing value across all features, were available for analysis. Participants were prompted to provide self-reports about their mental states (Table 2) every Monday, Wednesday, and Friday, with only a minority (3%) of self-reports being obtained on other days of the week. In each self-report survey, 10 distinct items asked the participant about a particular mental state over the recent past, as rated on a 4-point ordinal scale (“Not at all”, “A little”, “Moderately”, “Extremely”). There were 5 positive items for which a high score describes a positive outcome, and 5 negative items for which a high score describes a negative outcome. A total of 6,364 surveys were completed, corresponding to 63,640 mental state items being rated.

**Table 1:** Passive sensing data.

Features	Source
Duration of detected physical activity, informing on sedentary behavior and modes of transportation (in vehicle, on bike, on foot, walking, running, still, tilting, unknown).	Accelerometer
Sleep patterns: start time/ end time and sleep duration.	Accelerometer, light sensor, microphone
Location: the distance travelled and number of distinct locations visited were computed.	GPS
Phone usage: lock/unlock frequency and duration as well as the number and duration of incoming or outgoing calls, missed calls or SMS, which may be indicative of social interactions but not their content.	Phone metadata as well as call and SMS logs
Number and duration of ambient conversations, which inform on the presence of people around the phone owner but does not necessarily involve his/her active participation.	Microphone
Time: day of the week, day of the month, weekend (yes/no) which reflect important cycles that structure our lives.	Clock

High-level features available in the CrossCheck dataset come from various sources. Features extracted from sensors were indicative of physical activity, sleep patterns, mobility and social interactions, among other things. Features were computed separately for multiple consecutive 6-hour periods.

**Table 2:** Surveys.

Item name	Item question: Have you been…	Valence	Lower	Higher
Calm	feeling calm	Positive	0, 1, 2	3
Hopeful	hopeful about the future	Positive	0, 1, 2	3
Sleep	sleeping well	Positive	0, 1, 2	3
Social	social	Positive	0, 1	2, 3
Think	able to think clearly	Positive	0, 1, 2	3
Depressed	depressed	Negative	0	1, 2, 3
Harm	worried about people trying to harm you	Negative	0	1, 2, 3
Seeing things	seeing things other people can’t see	Negative	0	1, 2, 3
Stressed	feeling stressed	Negative	0	1, 2, 3
Voices	bothered by voices	Negative	0	1, 2, 3

10 types of mental states were self-reported on a 4-point ordinal rating scale (Classes/labels: “Not at all” = 0, “A little” = 1, “Moderately” = 2, “Extremely” = 3). High scores indicated a positive outcome for 5 items (e.g., Calm) and a negative outcome for the 5 others (e.g., Depressed). Recoding the original ordinal labels into binary classes consisted in contrasting 1 class against the 3 others, except for one mental state (Social).

### Data processing

**Fig 1:**
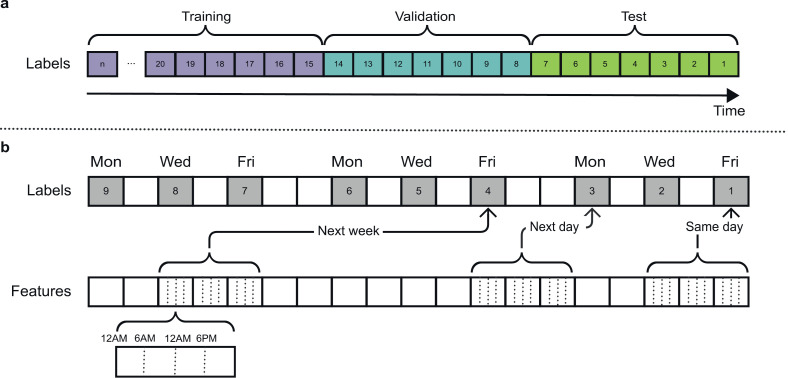
Time-based splitting strategy and forecast horizons. **a.** The test set contained the latest 7 surveys from each participant, the validation set contained the previous 7 surveys, and the training set included all (>7) earlier surveys **b.** Each label to predict was paired with 3 days of input data (12 6-hour periods), separately for 3 forecast horizons (same day, next day, next week).

The dataset was partitioned into time-based training, validation and test sets that accounted for temporal dependencies in the data [43,52]. The test set contained the latest 7 surveys from each participant, the validation set contained the previous 7 surveys, and the training set included all (>7) earlier surveys (Fig 1). Participants with less than 21 surveys in total were excluded. Consequently, the number of participants decreased from 62 to 61 for models predicting the next week horizon. Depending on the forecast horizon, the training set included from 5,163 to 5,307 surveys while the validation and test sets each included 427 to 434 surveys. By representing each participant equally in the test set, model evaluation was not biased by participants contributing more data. As a trade-off, the number of training examples per participant varied importantly from 9 to 181 (median = 90.5, interquartile range = 74.5). The training and validation sets were used for model development (preliminary experiments, hyperparameter tuning, etc.) while the test set served for final model performance evaluation. Since our splitting strategy does not control for distribution drift [53,54], we assessed distribution variation across time splits, especially for rarer classes.

Since the CrossCheck dataset includes high-level features extracted from raw sensor data, our preprocessing pipeline primarily served to ensure the XGBoost [55] and LSTM [56] models received equivalent input information while meeting their respective requirements. After dataset splitting, features were standardized at the group level to Gaussian-like distributions using the Yeo-Johnson method [57]. While tree-based methods such as the XGBoost algorithm are insensitive to scaling transformations [58], this preprocessing step helps LSTM models converge [59]. For LSTM models, each self-report was paired with a sequence of 3 consecutive days divided in 6-hour periods of passive sensing data. The 3-day input sequence creation method leads to some days being present twice in the input data, and some days of the week being over-represented. The duplicated days depend on the forecast horizon used to create sequences. For XGBoost models, input sequences were reshaped into tabular format.

### Forecasting

We aimed to predict future self-reported mental states using passive smartphone data exclusively. Past self-reports were not included in the models’ input contrary to predictive models described in some previous work [31–33]. In total, 120 distinct machine learning models were trained. We forecasted 10 mental states (Table 2) over 3 forecast horizons (same day, next day, next week) with 2 machine learning algorithms (XGBoost, LSTM) on 2 predictive tasks (ordinal regression, binary classification). The forecast horizon was the time gap between the input data and the predicted label, which increased from 0 day to 1 day and 7 days (Fig 1). XGBoost and other gradient-boosted decision trees algorithms were successful for regression of current day and future self-report aggregates [45,48], and future clinical scale ratings [51] on Crosscheck data. LSTM models and other recurrent neural networks have also provided accurate forecasts of mood and stress in healthy subjects [31,32] and of depressive states in self-identified depressed individuals [33].

### Learning task

#### Ordinal regression.

Like multiclass classification, ordinal regression involves multiple discrete classes, and like continuous regression, it considers an ordering of values. To predict values from (0, 1, 2, 3) corresponding to “Not at all”, “A little”, “Moderately” and “Extremely”, our XGBoost implementation simultaneously learned a continuous regression task and tuned the default thresholds (0.5, 0.15, 2.5) to discretize the continuous predicted values into (0, 1, 2, 3). For LSTM models, we used the Conditional Ordinal Regression for Neural networks approach and their open source implementation in *coral-pytorch* [39]. The neural network architecture allows a single model to decompose the ordinal regression task of predicting values (0, 1, 2, 3) into 3 independent binary tasks of predicting >0, >1, and >2. XGBoost and LSTM models were respectively trained using the regression squared loss and the conditional ordinal regression loss for neural networks. The performance of both model types was optimized for the macro-averaged mean absolute error (MAMAE), which is robust to class imbalance [60] observed in the CrossCheck dataset. This metric computes the mean absolute error (MAE) per class then averages results, giving equal weight to each class (S1 Text). For the sake of comparison with previous works, models were also evaluated using regular MAE which does not appropriately handle class imbalance [18].

#### Binary classification.

**Fig 2:**
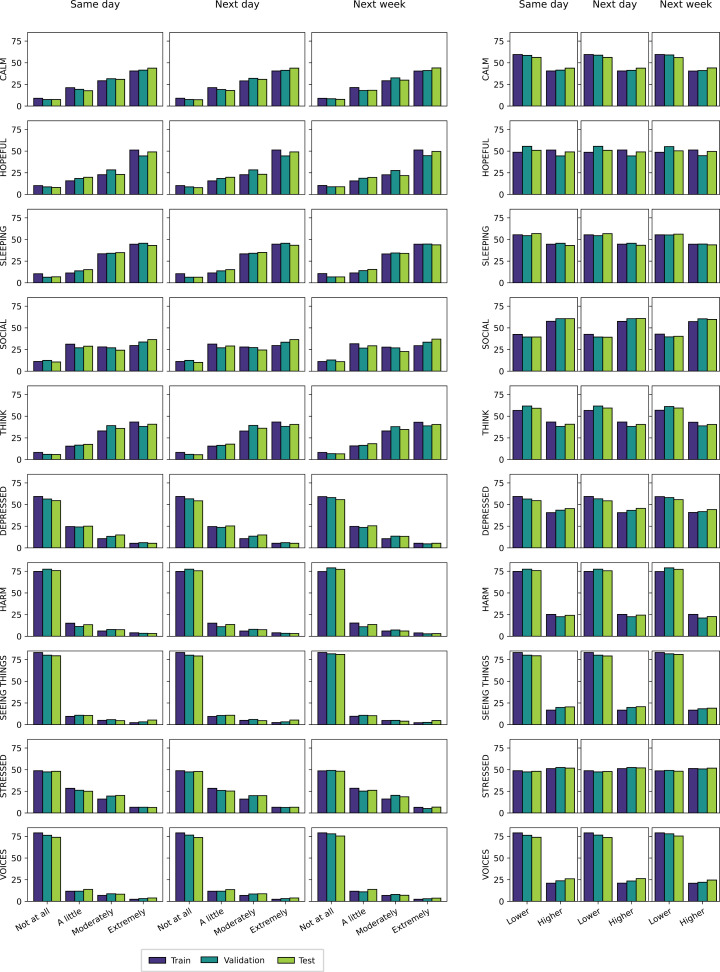
Label distributions across mental states and dataset splits. The left and right panels show the distributions of the original ordinal labels and recoded binary labels, respectively. Each row is associated with a mental state and each inner column with a forecast horizon. Bar plots display the proportion (%) of examples for the 4 or 2 classes (labels) in each dataset split.

The original 4 classes (“Not at all”, “A little”, “Moderately” and “Extremely”) were binarized using the cutoff resulting in the two best-balanced classes. Due to the skewed nature of the original label distributions, this consisted in contrasting one class against the other 3, except for one variable. Specifically, “Extremely” was converted to “Higher” and other labels to “Lower” for positive items, while “Not at all” was converted to “Lower” and all other labels to “Higher” for negative items. Due to a flatter distribution, the variable *Social* had “Extremely” and “Moderately” converted to “Higher” and the other two values to “Lower”. Binary classes remained imbalanced to some degree, very much so in some instances (Fig 2). Both XGBoost and LSTM models were trained using the binary cross entropy loss and evaluated with balanced accuracy (BAcc) to deal with class imbalance [61–63]. BAcc is the arithmetic mean of sensitivity (true positive rate) and specificity (true negative rate). For the sake of comparison with a large part of the relevant literature [30], models were also evaluated using regular accuracy (Acc).

### Model training and validation

For each XGBoost model, the development phase included an automated 10 rounds of hyperparameter optimization using the Optuna library with the TPESampler over the train and validation sets [64]. Once the best hyperparameters were determined, the final model was trained on both the train and validation sets and evaluated on the test set. For each LSTM model, the PyTorch Lightning *Tune* functionality was used at the beginning of training to find the optimal learning rate and batch size [65]. Given the important computational cost of searching the hyperparameter space, a partial grid search was conducted along with a manual inspection of training and validation curves. The following hyperparameters were selected for achieving good generalization across mental states, forecast horizon, and task: 150 epochs and 1 LSTM layer with 128 nodes and 10% dropout. While this architecture may be considered to have lower capacity, it is well in line with previous relevant works [31–33].

### Statistical testing

Statistical analyses were conducted to assess if models performed significantly above baseline and compare them across algorithms, forecast horizons, mental states, and learning tasks. For each of the 60 conditions (2 tasks, 3 forecast horizons, 10 mental states), a baseline distribution of performance scores (MAMAE or BAcc) was generated using a Monte Carlo method. To build a performance distribution, 1,000 prediction samples were drawn with replacement from the training set and evaluated against the test set (S2 Text). This baseline represents “best guess” predictions based on previous mental state self-reports and constitute a more challenging baseline than random/chance-level predictions. A model significantly outperformed the baseline if its test performance was better than the baseline quantile corresponding to a *p value* <.05 with a Bonferroni correction for 120 models (*p* < 4.2 x 10^−4^). Considering ordinal regression and binary classification tasks separately, model performances on the test set were compared between the XGBoost and LSTM algorithms using the non-parametric Wilcoxon signed-rank test and across forecast horizons (3 horizons) and mental states (10 variables) with non-parametric Friedman tests [52,66]. To compare performance between the ordinal regression and binary classification tasks, the MAMAE and BAcc values were transformed to a scale-normalized balanced error which ranges from 0 to 1 (S1 Text). The relationship between the scale-normalized balanced error of ordinal and binary models was compared to a perfect correlation and the residuals were inspected for discrepancies between the two learning tasks. Finally, the effect of class imbalance was assessed using the Spearman rank correlation between the predictive performance and the class imbalance of each of the 10 mental states. Class imbalance was quantified as the difference between the number of examples of the majority and the minority class, normalized by the total number of examples (0 to 1 range). The same definition was applied for ordinal regression and binary classification.

## Results

### Descriptive statistics

Participants tended to rate more frequently high (“Extremely”) on positive items (Calm, Hopeful, Sleeping, Social, Think) and low (“Not at all”) on negative items (Depressed, Harm, Seeing Things, Stressed, Voices) (Fig 2). With 76% to 81% of labels belonging to “Not at all” and only 3% to “Extremely”, the class imbalance for the negative items Harm, Voices and Seeing Things was major. A lesser but still considerable imbalance between majority (33% to 48%) and minority (7 to 11%) classes was observed for positive items. Recoding ordinal classes into binary classes reduced imbalance, yet it remained very large in some cases (Harm, Voices and Seeing Things) with 76–81% for the majority class (“Lower”) and only 19–24% for the minority class (“Higher”). Upon visual inspection, no notable distribution shifts were observed between the training, validation and test sets created via time-based splitting (Fig 2). The negligible variation between splits suggests they are representative of the full dataset and the validation split will properly estimate test performance.

### Forecasting performance

**Fig 3:**
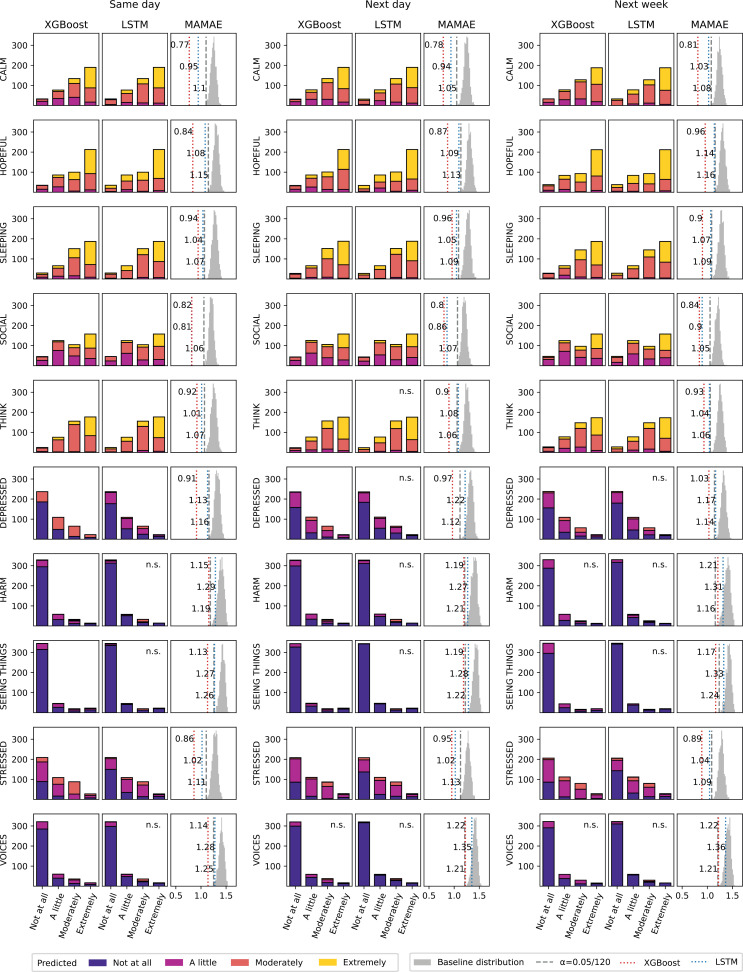
Ordinal regression task performance. Each row is associated with a mental state and each outer column with a forecast horizon. For each of the 30 conditions, bar plots show XGBoost and LSTM test predictions. The bar height corresponds to the number of examples with each bar indicating the true class and colors reflecting the predicted classes. The MAMAE for these XGBoost and LSTM model predictions are shown against the MAMAE significance threshold (Bonferroni corrected *P* < .05) and the corresponding Monte Carlo baseline distribution. n.s., non-significant.

For ordinal regression, 45 out of 60 models across algorithms, forecast horizons and mental states performed significantly above baseline (Bonferroni corrected *P* < .05) (Fig 3, S1 Fig). Non-significant models were associated with negative mental states (Depressed, Harm, Seeing things, Stressed, Voices) and the LSTM algorithm. MAMAE values ranged from 1.36 to 0.77 (median = 1.04) across all models, with the 45 significant models not exceeding MAMAE = 1.19 (median = 0.96). There was a significant difference in performance between the 2 algorithms (*Z* = 1, *P* < .001) with XGBoost models (median = 0.94) performing better than LSTM models (median = 1.08). Similarly, a significant effect of forecast horizon was observed (*Q* = 19.9, *P* < .001) although the decrease in performance as the horizon increased from same day (median = 1.02) to next day (median = 1.03) then next week (median = 1.04) was very small.

**Fig 4:**
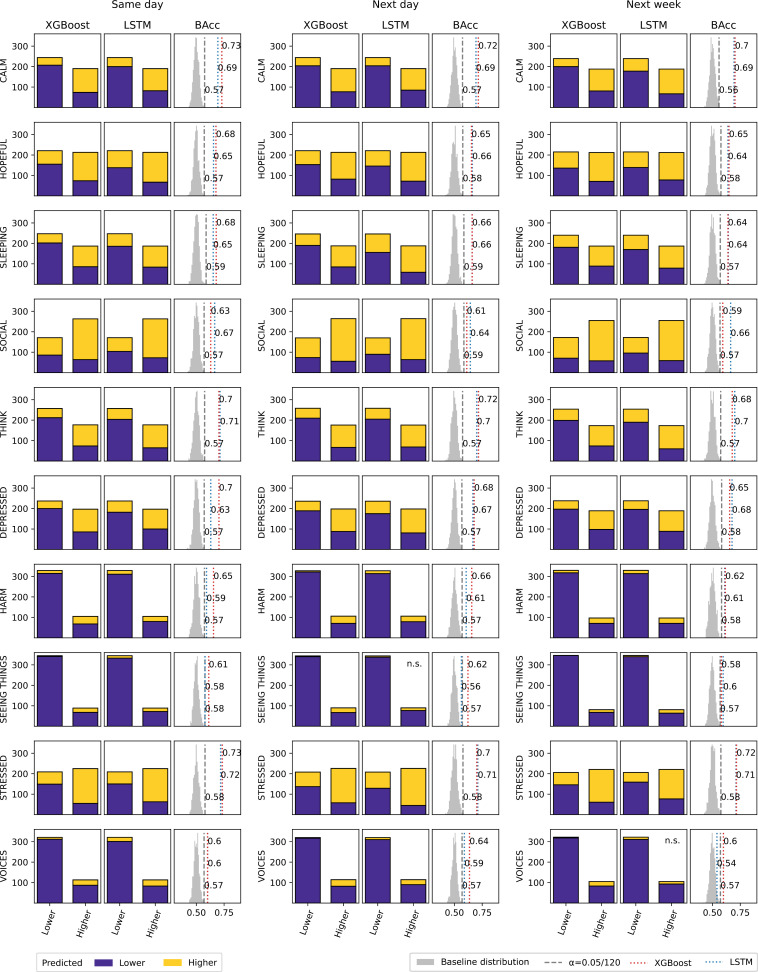
Binary classification task performance. Each row is associated with a mental state and each outer column with a forecast horizon. For each of the 30 conditions, bar plots show XGBoost and LSTM test predictions. The bar height corresponds to the number of examples with each bar indicating the true class and colors reflecting the predicted classes. The BAcc for these XGBoost and LSTM model predictions are shown against the BAcc significance threshold (Bonferroni corrected *P* < .05) and the corresponding Monte Carlo baseline distribution. n.s., non-significant.

**Fig 5:**
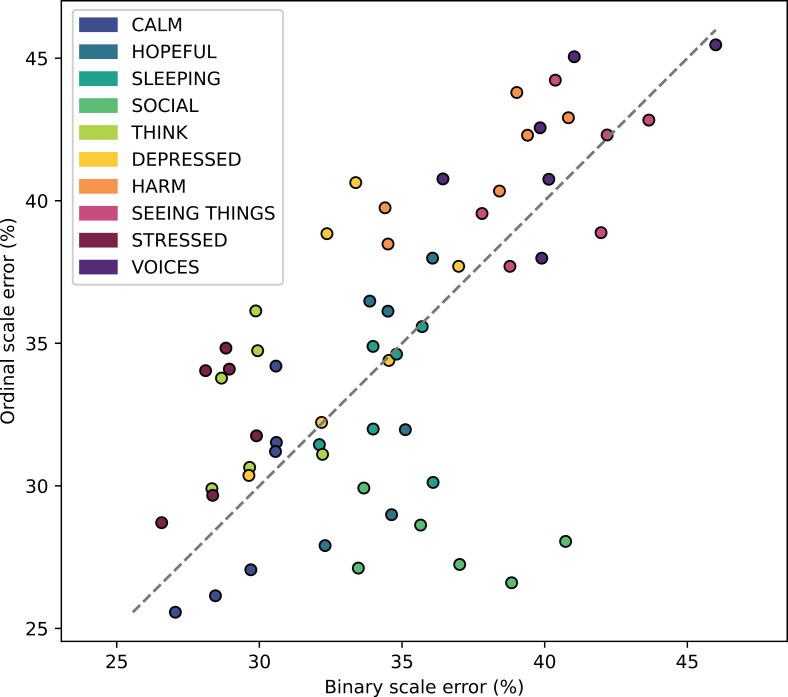
Relationship between ordinal regression and binary classification performance. For each of the 60 conditions (10 mental states x 2 algorithms x 3 forecast horizons), the performance of the ordinal regression model (y-axis) is displayed against the binary classification model (x-axis) under the same condition. Balanced performance metrics (MAMAE, BAcc) were normalized to a common scale ranging from 0 to 1 (see S1 Text).

On the binary classification task, 58 out of 60 models were significantly superior to baseline (Bonferroni corrected *P* < .05) (Fig 4, S2 Fig). BAcc values ranged from 54% to 73% (median = 66%) with significant models all performing above 58%. Contrary to ordinal regression, no significant difference in performance was found (*Z* = 150, *P* = .1) between the XGBoost (median = 66%) and LSTM (median = 66%) algorithms. A significant effect of the forecast horizon was detected (*Q* = 7.9, *P* = .02). The decrease in performance over same day (median = 66%), next day (median = 66%), and next week (median = 65%) was consistent with the effect observed for ordinal regression. When comparing the scale-normalized balanced error of ordinal regression and binary classification to a perfect correlation, the very low average of residuals (= 0.003) suggests equivalent performance on the two tasks on average, with no predictive task clearly outperforming the other (Fig 5).

### Class imbalance

**Fig 6:**
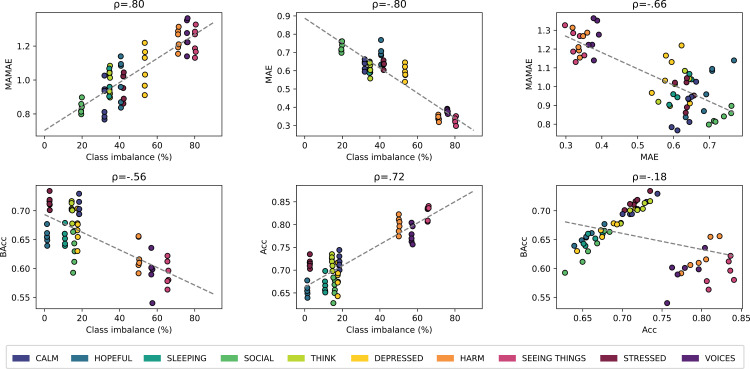
Effect of class imbalance on predictive performance. Each scatter plot displays the test performance of 60 models under different conditions (10 mental states x 2 algorithms x 3 forecast horizons). Rows show the effect of class imbalance on performance for ordinal regression (top) and binary classification (bottom). The left and middle columns respectively highlight the relationship between class imbalance and predictive performance for balanced and unbalanced metrics, and the right column reveals the relationship between the two metrics. Each correlation is quantified by Spearman’s ρ (rho).

Very strong effects on performance were unravelled for mental states, both for ordinal regression (*Q* = 49.4, *P* < .001) and binary classification (*Q* = 47, *P* < .001) (Figs 3 and 4). Further inspection revealed that the effect of mental states could be explained by their class imbalance, as it correlated with MAMAE values of ordinal regression (Spearman r = .8) and BAcc for binary classification (r = −.56) (Fig 6). Large class imbalance observed for the variables Harm, Voices and Seeing Things, was associated with poor performance (high MAMAE, low BAcc). In stark contrast, opposite effects were observed when using metrics that do not account for class imbalance (MAE, Acc). Indeed, large class imbalance was associated with higher performance, both for the ordinal regression MAE (r = −.8) and the binary classification Acc (r = .72). As a corollary, performance metrics that do and do not account for class imbalance should be negatively correlated, which held for MAMAE and MAE (r = −.66) as well as BAcc and ACC (r = −.18). The latter association was weaker given 7 out of 10 mental states were fairly balanced and their BAcc and Acc strongly positively correlated (r = .95).

## Discussion

We show that ordinal regression, which best preserves key clinical information, can forecast self-reported mental states from passive smartphone data with predictive performance levels comparable to those of binary classification. Class imbalance, which may be particularly pronounced for ordinal data, strongly affects model training and performance is inadequately rendered when unfit evaluation metrics are used. The XGBoost performs as well or even better than the LSTM algorithm for forecasting. Increasing the forecast horizon incurred a negligible decrease in performance.

While mental states are often collected on ordinal rating scales in digital phenotyping research, the majority of past studies formulated predictions using binary classification [24,26,32] or continuous regression [29,48,51]. Ordinal models adequately preserve the order of discrete classes without assuming continuity between them. Importantly, ordinal predictions exist on the data collection scale and can be interpreted by clinicians using the scale’s interpretation guidelines. Critically, our findings demonstrate that using an ordinal regression modelling that best meets this clinical motivation does not lead to any systematic cost in predictive performance compared to using binary classification.

The rarity of clinically relevant mental-health events (e.g., psychotic episode) leads to datasets composed mainly of healthy examples, both in terms of inputs and labels. In this study, mental states with larger class imbalance (e.g., Harm, Voices, Seeing things) were associated with lower performance, a typical challenge in machine learning [67]. Since imbalance is a core property of mental health data, it is best to leave it unadjusted and use adapted methods [63]. The majority of past digital phenotyping studies only report performance metrics that do not account for class imbalance (e.g., Acc, root mean squared error, MAE) [30], thereby allowing models that predict only the majority class to appear deceptively good. To overcome these issues, we used metrics that account for class imbalance (BAcc and MAMAE) to train and evaluate models. We show that models appear to perform best on variables describing rare events (e.g., Harm, Voices, Seeing things) when using unbalanced metrics, but that relationship is reversed when using the adequate balanced metrics. Consequently, inappropriate metrics can lead to systematically incorrect conclusions when evaluating model performance or the impact of procedures such as feature engineering, feature selection, or model selection. Besides, resampling techniques have been used in past digital phenotyping studies in an attempt to improve performance by mitigating class imbalance [68]. However, these methods provide little to no benefits for modern algorithms like XGBoost [69]. Instead, tuning the model’s decision threshold is a more sensible approach. Resampling provides no performance improvement for binary models of medical diagnosis and deteriorates model calibration, which should be a key performance criterion when a probabilistic interpretation is necessary (e.g., risk scores) [70]. Cost-sensitive learning, which incorporates the outcome of a prediction into the learning process, has also been shown to be an effective solution to overcome class imbalance [71].

Recurrent neural networks, in particular the LSTM approach, are specialized for sequence data such as sensor data [36,37] and are popular forecasting algorithms in digital phenotyping [32,33,35,72]. On the other hand, gradient-boosted decision trees have been consistently successful in diagnostic studies [45,51,73,74], but few investigated them for forecasting [48,75]. Our results show that XGBoost models are equally capable as LSTM models for forecasting, and even superior under certain conditions. Gradient boosted decision trees were previously found to be superior to neural networks on tabular datasets with skewed features, uninformative features, or rare classes [76,77], all typical characteristics of digital phenotyping. Given similar levels of performance, algorithms should be selected according to other practical implications such as explainability, the amount of required training data, or computational costs.

Increasing the forecast horizon from “same day” to “next week” was associated with a negligible performance degradation, in line with previous findings [31,34]. Given the strong weekly seasonality in behavior and mental health self-reports [31,33], one should proceed with care when extrapolating performance to different days of the week. For instance, the CrossCheck study collected self-reports primarily on Monday, Wednesday, and Friday, limiting our ability to train more robust models. Besides, increasing the input history length up to 3 weeks could improve model performance [31–33,48,68,78], given early warning signs have been observed up to 30 days prior to symptom worsening in mental illness [47,79,80].

Although the levels of forecast performance we achieved are encouraging, predictions are not sufficiently accurate to consider their implementation in the clinical realm. Furthermore, calibrating and evaluating model performance on data from various contemporary smartphone devices with differing sensor quality is a crucial step before any applied use of the predictive models we describe. Future work is thus required to significantly improve the performance of forecast models using digital phenotyping data, whether by exploiting much larger datasets, improving feature engineering, or redefining more optimal clinical targets, among other things. For instance, different mental states can be correlated [68] and modelling techniques able to leverage this information can improve forecasting performance [29]. Multiple-output methods are possible for both tree-based and deep-learning models, but the intersection with ordinal regression methods has yet to be investigated.

Furthermore, it will be key to explain why a given model makes a specific prediction, a critical subject the present study did not explore. Explainable machine learning systems will indeed be necessary for clinicians to be able to decide whether to trust or not a prediction and to comply with regulations in some jurisdictions [81]. A popular technique is to attribute SHapley Additive exPlanations (SHAP) values [82] to determine the contribution of each feature towards a prediction. However, Shapley additive explanations can be misleading since they are detached from prediction certainty. Poorer model calibration associated with imbalanced data decreases their reliability. The *calibrated explanations* method [83] produces probabilistic prediction intervals and scores each feature based on its contribution towards the prediction and its uncertainty. Empirical studies showed that predictions and explanations need to be grounded in the users’ task and fit their mental model to be useful [84]. For digital phenotyping, this means explanations would benefit from higher-level features that relate to mental health constructs instead of features closer to raw smartphone data, which may be at odds with improving predictive performance.

Our uniform methodology was applied to train and evaluate 120 models and provide a fair and comprehensive benchmark. As a trade-off, there might be better achievable performance on each individual task. For instance, the public version of the CrossCheck dataset was used without further feature engineering or selection. Adding well-crafted features could have benefited XGBoost and LSTM models unevenly since neural networks are more sensitive to uninformative features. On another note, we only investigated group models. Each participant was represented equally in the test set to prevent the number of self-reports biasing the evaluation. However, participants with more training examples may indirectly bias results since per-person models typically perform better [18,45,51].

The unprecedented volume and richness of data about the individual generated by smartphones opens a unique window onto mental health. Beyond the precise monitoring of psychiatric conditions in everyday life, digital phenotyping data paired with machine learning models allows to forecast future mental states. Accurate prediction of the likely progression of the illness would be key to implementing personalized prevention measures. While research in this field is burgeoning, issues remain to be addressed before such forecast models can be implemented as clinical decision support tools. In this study, we explored modelling approaches that preserve the maximum of clinical information from the collected ordinal data by training ordinal regression models instead of binary classification models to forecast mental states. Importantly, we show that the clinically motivated ordinal approaches do not incur a trade-off in predictive performance. Given class imbalance is challenging for learning algorithms and is a core property of mental health data, we argue that using binary classification is an unsatisfactory mitigation method and using evaluation metrics that account for its impact is essential. Finally, we question recurrent neural networks as the *de facto* superior forecast algorithm and thus encourage a more systematic benchmarking of machine learning algorithms, especially gradient boosted decision trees, to predict future mental states using digital phenotyping data.

## Supporting information

S1 Text Metric definitions. Mathematical formulas for the binary classification and ordinal regression metrics. It includes the procedure to compute scale-normalized balanced error and MAMAE along with a proof and defines the class imbalance measure.
(PDF)

S2 Text Monte Carlo baseline distribution. Pseudocode to produce baseline distributions used in statistical tests.
(PDF)

S1 Fig Ordinal regression predictions. Each row is associated with a mental state and each outer column with a forecast horizon. For each of the 30 conditions, heatmaps show XGBoost and LSTM test predictions. The cell indicates the number of predictions with x-axis being the true class and the y-axis the predicted class. The color indicates the per-class recall value on the diagonal. This figure adds context to the Fig 3 and provides raw values to allow any metrics to be computed.
(TIF)

S2 Fig Binary classification predictions. Each row is associated with a mental state and each outer column with a forecast horizon. For each of the 30 conditions, heatmaps show XGBoost and LSTM test predictions. The cell indicates the number of predictions with x-axis being the true class and the y-axis the predicted class. The color scale is normalized per column, indicating the per-class recall value on the diagonal. This figure adds context to the Fig 4 and provides raw values to allow any metrics to be computed.
(TIF)
