# Supplementary material for: Forecasting mental states in schizophrenia using digital phenotyping data
Source: PLOS Digit Health. 2025 Feb 7;4(2):e0000734. doi: 10.1371/journal.pdig.0000734 (PMC11805420; doi:10.1371/journal.pdig.0000734)
Supplement: S1 Text — Mathematical formulas for the binary classification and ordinal regression metrics. It includes the procedure to compute scale-normalized balanced error and MAMAE along with a proof and defines the class imbalance measure. (PDF) [file pdig.0000734.s001.pdf]

S1 Appendix. Metric definitions

Definitions

- TP: True positive
- TN: True negative
- FP: False positive
- FN: False negative
- i: Example index
- j: Class index
- k: Number of classes
- x_i: Input
- y_i: True label
- f: Predictor
- $f(x_i)$: Predicted label
- S: Set of all examples
- S_j: Set of examples of class j

Binary classification metrics

$$\text{Accuracy (Acc)} = \frac{\text{TP} + \text{FN}}{\text{TP} + \text{FP} + \text{TN} + \text{FN}}$$

$$\begin{aligned}\text{Balanced accuracy (BAcc)} &= \frac{1}{2}\left(\frac{\text{TP}}{\text{TP} + \text{FN}} + \frac{\text{TN}}{\text{TN} + \text{FP}}\right) \\ &= \frac{1}{2}\left(\text{sensitivity} + \text{specificity}\right) \\ &= \frac{1}{2}\left(\text{precision} + \text{recall}\right)\end{aligned}$$

$$\text{Scale normalized balanced error} = \frac{1 - \text{BAcc}}{k - 1} \qquad k = 2$$

Ordinal regression metrics

$$\text{Mean absolute error (MAE)} = \frac{1}{|S|} \sum_{x_i, y_i \in S} |f(x_i) - y_i|$$

$$\text{Macro-averaged mean absolute error (MAMAE)} = \frac{1}{k} \sum_{j=1}^{k} \frac{1}{|S_j|} \sum_{x_i, y_i \in S_j} |f(x_i) - y_i|$$

$$\text{Scale normalized MAMAE} = \frac{\text{MAMAE}}{k - 1}$$

Proof: Balanced error is equivalent to MAMAE for binary case

$$\begin{aligned}
\text{BAcc} &= \frac{1}{2}\left(\frac{\text{TP}}{\text{TP}+\text{FN}} + \frac{\text{TN}}{\text{TN}+\text{FP}}\right) && (1)\\
&= \frac{1}{2}\left(\frac{\text{TP}}{|S_1|} + \frac{\text{TN}}{|S_2|}\right) && (2)\\
&= \frac{1}{2}\left(\frac{\sum_{x_i\in S_1} 1_{\{f(x_i)=1\}}}{|S_1|} + \frac{\sum_{x_i\in S_2} 1_{\{f(x_i)=2\}}}{|S_2|}\right) && (3)\\
&= \frac{1}{2}\sum_{j=1}^{2}\left(\frac{\sum_{x_i\in S_j} 1_{\{f(x_i)=j\}}}{|S_j|}\right) && (4)\\
&= \frac{1}{2}\sum_{j=1}^{2}\frac{1}{|S_j|}\sum_{x_i\in S_j} 1_{\{f(x_i)=j\}} && (5)\\
&= \frac{1}{2}\sum_{j=1}^{2}\frac{1}{|S_j|}\sum_{x_i\in S_j} (1 - 1_{\{f(x_i)\neq j\}}) && (6)\\
&= \frac{1}{2}\sum_{j=1}^{2}\frac{1}{|S_j|}\sum_{x_i\in S_j} 1 - \frac{1}{2}\sum_{j=1}^{2}\frac{1}{|S_j|}\sum_{x_i\in S_j} 1_{\{f(x_i)\neq j\}} && (7)\\
&= \frac{1}{2}\sum_{j=1}^{2}\frac{1}{|S_j|}|S_j| - \frac{1}{2}\sum_{j=1}^{2}\frac{1}{|S_j|}\sum_{x_i\in S_j} 1_{\{f(x_i)\neq j\}} && (8)\\
&= 1 - \frac{1}{2}\sum_{j=1}^{2}\frac{1}{|S_j|}\sum_{x_i\in S_j} 1_{\{f(x_i)\neq j\}} && (9)\\
&= 1 - \frac{1}{2}\sum_{j=1}^{2}\frac{1}{|S_j|}\sum_{x_i\in S_j} |f(x_i) - y_i| \qquad y \in \{0,1\} && (10)\\
&= 1 - \frac{1}{k}\sum_{j=1}^{k}\frac{1}{|S_j|}\sum_{x_i\in S_j} |f(x_i) - y_i| \qquad k = 2 && (11)\\
&= 1 - \text{MAMAE} && (12)\\
1 - \text{BAcc} &= \text{MAMAE} && (13)
\end{aligned}$$

Class imbalance

$$\begin{aligned}
S_{maj} &= \max\left(\{|S_1|, |S_2|, \ldots, |S_k|\}\right)\\
S_{min} &= \min\left(\{|S_1|, |S_2|, \ldots, |S_k|\}\right)\\
\text{class imbalance} &= \frac{|S_{maj}| - |S_{min}|}{k-1}
\end{aligned}$$
