# Supplementary material for: Forecasting mental states in schizophrenia using digital phenotyping data
Source: PLOS Digit Health. 2025 Feb 7;4(2):e0000734. doi: 10.1371/journal.pdig.0000734 (PMC11805420; doi:10.1371/journal.pdig.0000734)
Supplement: S2 Text — Pseudocode to produce baseline distributions used in statistical tests. (PDF) [file pdig.0000734.s002.pdf]

S2 Appendix. Monte Carlo baseline distribution

Procedure

```
k = 1000  # number of sampling iterations
train = [...]  # array of training labels
test = [...]  # array of test labels

metric_distribution = [ ]  # array of sample metrics
for i = 1 to k:
    predictions_sample = []  # array of sampled predictions

    # draw a prediction from ‘train’ with replacement
    for j = 1 to len(test):
        naive_prediction = sample_with_replacement(train)
        predictions_sample.append(naive_prediction)

    # calculate metric from ‘test‘ and "naive" predictions
    sample_metric = f(test, predictions_sample)
    distribution.append(sample_metric)

return metric_distribution
```
