# Supplementary figures and images for: Forecasting mental states in schizophrenia using digital phenotyping data
Source: PLOS Digit Health. 2025 Feb 7;4(2):e0000734. doi: 10.1371/journal.pdig.0000734 (PMC11805420; doi:10.1371/journal.pdig.0000734)

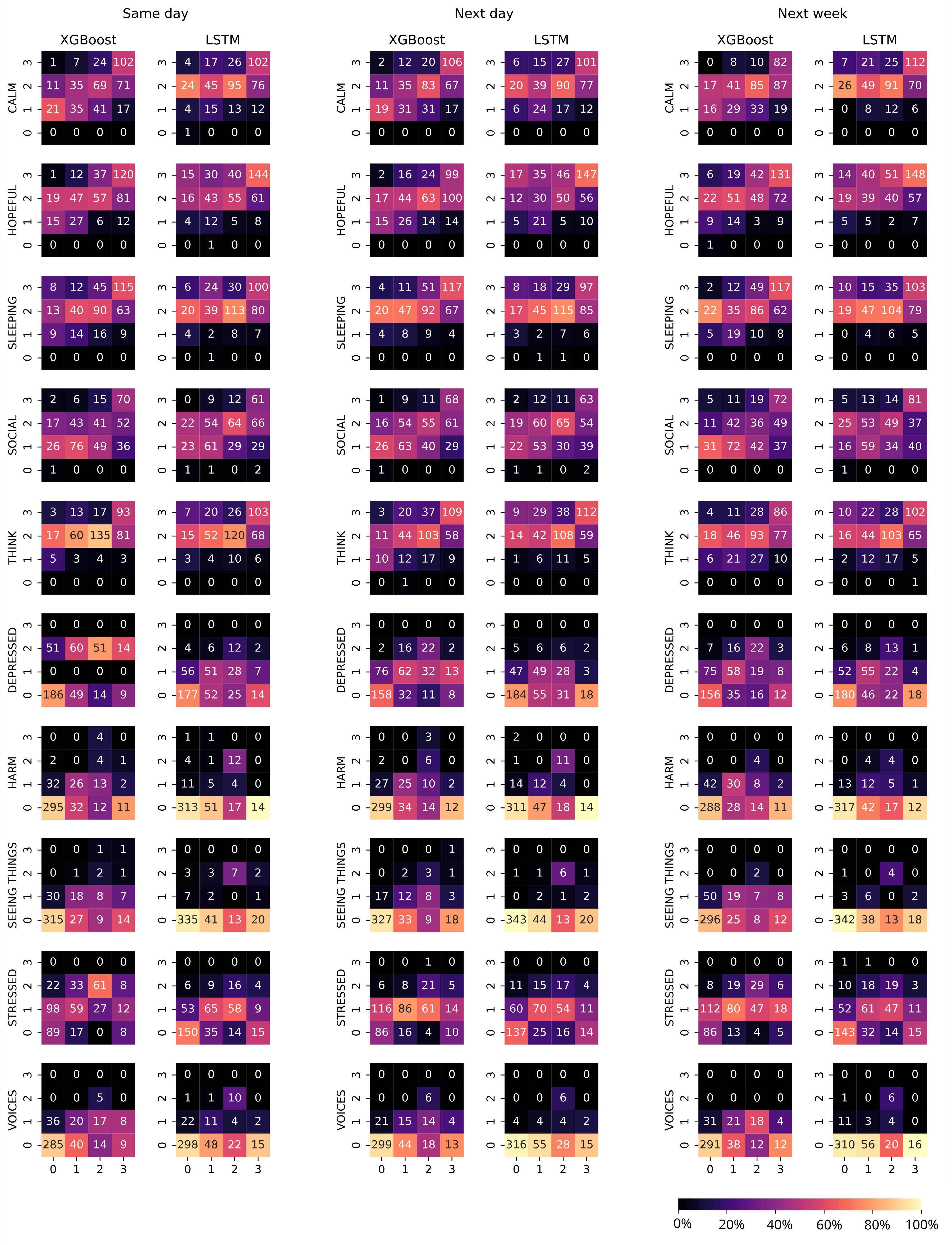

Supplement: S1 Fig — Each row is associated with a mental state and each outer column with a forecast horizon. For each of the 30 conditions, heatmaps show XGBoost and LSTM test predictions. The cell indicates the number of predictions with x-axis being the true class and the y-axis the predicted class. The color indicates the per-class recall value on the diagonal. This figure adds context to the Fig 3 and provides raw values to allow any metrics to be computed. (TIF) [file pdig.0000734.s003.tif]

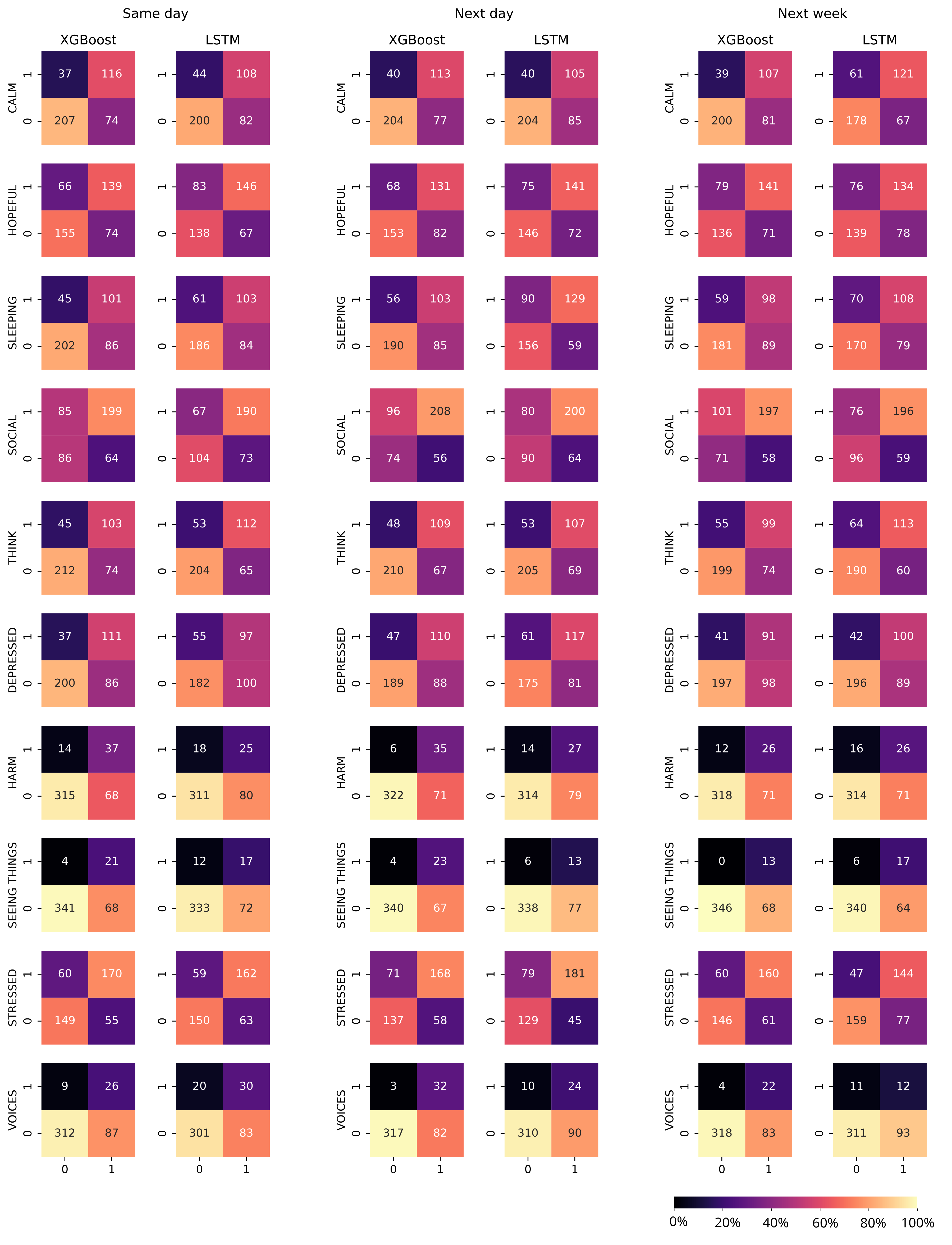

Supplement: S2 Fig — Each row is associated with a mental state and each outer column with a forecast horizon. For each of the 30 conditions, heatmaps show XGBoost and LSTM test predictions. The cell indicates the number of predictions with x-axis being the true class and the y-axis the predicted class. The color scale is normalized per column, indicating the per-class recall value on the diagonal. This figure adds context to the Fig 4 and provides raw values to allow any metrics to be computed. (TIF) [file pdig.0000734.s004.tif]
